# Draft Genome Sequences of Switchgrass Diazotrophs

**DOI:** 10.1128/MRA.00284-21

**Published:** 2021-05-27

**Authors:** Lauren B. Jones, Chi Myoung-Hwan, Venkatacha Lakshmanan, Ivone Torres-Jerez, Yuhong Tang, Maira Sparks, Nicole R. Shapiro, Kelly D. Craven, Michael K. Udvardi

**Affiliations:** a Noble Research Institute, LLC, Ardmore, Oklahoma, USA; b Department of Energy Joint Genome Institute, Berkeley, California, USA; University of Arizona

## Abstract

We report the draft genome sequences of five native nitrogen-fixing bacteria associated with roots of switchgrass isolated from the tallgrass prairies of Oklahoma. Nitrogen-fixing genes, including the *nif* cluster, are conserved across the Klebsiella and *Kosakonia* strains.

## ANNOUNCEMENT

Switchgrass (Panicum virgatum L.) is an intensely studied, warm-season perennial species that is being developed as a bioenergy crop for liquid fuels and other bioproducts (1). It generally requires application of synthetic fertilizer for maximal, sustained biomass yields. However, an alternative source of nitrogen is biological nitrogen fixation (BNF), in which associated, diazotrophic bacteria convert atmospheric nitrogen to ammonia for the plant. In order to study and potentially enhance plant-diazotroph interactions, five BNF bacteria were isolated from switchgrass roots in the tallgrass prairies of northern Oklahoma, in the United States (36.7689, −96.3904), during the summer growing season of 2010 and were grown on nitrogen-deficient medium as described previously (2, 3). Here, we present the draft genomes of these five switchgrass rhizoplane diazotrophs, including their nitrogen fixation (*nif*) genes and other genes.

Bacterial isolates were grown in 869 medium from a single colony (4). DNA was extracted using the DNeasy plant minikit (Qiagen, Inc., Germantown, MD). Two hundred nanograms of DNA was sheared to 300 bp using the LE220 focused ultrasonicator (Covaris, Inc., Woburn, MA) and size selected by double solid-phase reversible immobilization (SPRI) (Beckman Coulter, Brea, CA). The Illumina library creation kit (Kapa Biosystems, Wilmington, MA) was used for end repair, A tailing, ligation of Illumina adapters (IDT, Inc., San Jose, CA), and multiplexing following the manufacturer’s instructions. Libraries were quantified by quantitative PCR using the KAPA library quantification kit for Illumina. The pooled paired-ended library was prepared and sequenced at the U.S. Department of Energy (DOE) Joint Genome Institute (JGI) (USA) on an Illumina HiSeq 2500 sequencer using TruSeq sequencing-by-synthesis (SBS) kits, version 4, with a 2 × 150-bp indexed run recipe.

The resulting sequencing reads were quality control filtered and assembled into scaffolds with AllPaths-LG, version r46652 (5) and annotated with the NCBI Prokaryotic Genome Annotation Pipeline (PGAP) (6). Taxonomic classification was performed using the 16S rRNA sequences for BLASTn searches (7).

Two Kosakonia radicincitans (formerly Enterobacter) species (strains NFIX03 and NFIX09) and three Klebsiella quasipneumoniae species (strains NFIX19, NFIX42, and NFIX56) were identified. The genomic characteristics are listed in Table 1. The conserved *nif* cluster (*nifHDKBCENQSUVL*) required for nitrogenase biosynthesis and BNF activity is present in all five isolates. The *nif* transcriptional activator (*nifA*) is present in all Klebsiella strains but absent in the *Kosakonia* strains, suggesting different regulation of *nif* expression. The *amtB* and *glnAB* genes required for ammonia transport and utilization, respectively, are present in all five genomes. The *Kosakonia* strains have more plant colonization genes, including those involved in chemotaxis (*cheABRVWYZ*, *mcpBC*, and *motB*), mobility (*chpACDE* and *pilGHIJK*), and biofilm formation (*flgE*, *fliCF*, and *motA*), than the Klebsiella strains, which have no biofilm genes, only the *cheY* chemotaxis gene, and the *chpCDE* and *pilIJK* mobility genes, indicating that *Kosakonia* strains may be better colonizers of switchgrass.

**TABLE 1:** Genomic information and accession numbers for five nitrogen-fixing bacterial strains isolated from switchgrass roots^*a*^

Bacterial isolate^*b*^	Genome size (bp)	No. of scaffolds	Scaffold *N*_50_ (bp)	No. of sequencing reads	Total bp sequenced	Total no. of genes	G+C content (%)	GenBank accession no.	SRA accession no.
Kosakonia radicincitans NFIX03	5,597,196	34	288,533	6,831,996	1,024,799,400	5,357	53.99	FPJU00000000	SRX2122634
Kosakonia radicincitans NFIX09	5,483,348	14	3,027,540	6,152,934	922,940,100	5,280	54.03	FOHP01000000	SRX2122594
Klebsiella quasipneumoniae NFIX19	5,217,298	21	373,421	6,826,716	1,024,007,400	4,910	58.21	FOAU00000000	SRX2122607
Klebsiella quasipneumoniae NFIX42	5,292,309	21	531,623	6,097,090	914,563,500	5,034	58.08	FMVG00000000	SRX2122649
Klebsiella quasipneumoniae NFIX56	5,439,064	33	286,212	6,642,240	996,336,000	5,157	57.93	FPJQ00000000	SRX2122658

a Default parameters were used for all software unless otherwise specified.

b Strains were classified on the basis of ≥99% similarity with 16S rRNA genomic sequences.

The draft genome sequences of the five switchgrass isolates will serve as references to modify and improve the ability of the isolates to supply fixed nitrogen and improve plant growth for sustainable bioenergy production.

### Data availability.

The draft genome sequences from this study were deposited in DDBJ/ENA/GenBank and the raw sequencing reads in the NCBI Sequence Read Archive (SRA). The corresponding GenBank and SRA accession numbers are listed in Table 1.
